# Southward spreading of the Changjiang Diluted Water in the La Niña spring of 2008

**DOI:** 10.1038/s41598-020-79634-y

**Published:** 2021-01-11

**Authors:** Chen-Tung Arthur Chen, Yan Bai, Ting-Hsuan Huang, Xianqiang He, Hsien-Wen Chen, Shujie Yu

**Affiliations:** 1 Department of Oceanography, National Sun Yat-Sen University, Kaohsiung, 804 Taiwan; 2 Institute of Marine Chemistry and Environment, Zhejiang University, Zhoushan, 316021 China; 3 State Key Laboratory of Satellite Ocean Environment Dynamics, Second Institute of Oceanography, Ministry of Natural Resources, Hangzhou, 310012 China; 4 Taiwan Ocean Research Institute, National Applied Research Laboratories, Kaohsiung, 801 Taiwan; 5 Department of Maritime Police, Central Police University, Taoyuan, 33304 Taiwan

**Keywords:** Climate sciences, Ocean sciences

## Abstract

The La Niña of 2007/2008 was particularly strong, so was the southward flow of the cold, nutrient-rich Changjiang (Yangtze River) Diluted Water (CDW) when the winter monsoon started to blow in the fall. Here we use shipboard data in 2008 in two transects, one in the southwestern East China Sea and one in the southern Taiwan Strait, to show that as late as April in 2008 the CDW was still clearly identifiable when the winter monsoon had weakened. Waters as cold as 16 °C with a salinity lower than 30 still occupied the southwestern East China Sea. Waters of 17 °C and S < 32 could also be found off the coast of China in the central Taiwan Strait. The concentration of NO_3_ + NO_2_ was higher than 18 μmol L^−1^ at both places, which was as much as 40 times higher than the northward moving South China Sea (SCS) water to the east. As a result, the Changjiang River plume may be a significant source of nutrients, particularly N, to the oligotrophic, N-poor SCS, especially in the La Niña years. Indeed, colder and more turbid CDW was more intense and went farther south in 2008 compared with the normal springs of 2006, 2007 and 2009.

## Introduction

The East China Sea (ECS) is among the largest marginal seas in the world and is one of the most productive areas of the world's oceans. One of the largest rivers in the world, namely the Changjiang (Yangtze) River empties into the shelf with large nutrient inputs^1–4^. Numerous studies have concentrated on the nutrient dynamics, eutrophication, and hypoxia on the shelf by following the Changjiang River Plume or the Changjiang Diluted Water (CDW).

Because the Changjiang River discharges 75% of its annual outflow in late spring–summer the CDW is the most intensive and spreads out the most in summer^5–10^. Further, because of the southwest monsoon in summer the CDW spreads northeastward and can be detected off the coasts of southeast Korea and southwest Japan. Part of the plume even enters the Sea of Japan/East Sea. As the CDW relates to the transport of freshwater, nutrients, pollutants, and coastal biological species to a wide area it has attracted much attention^11–17^.

On the other hand, in late fall-early spring when the northeast monsoon prevails the CDW spreads southward and is confined to a much smaller area hugging the coast of China^18–21^. This southward moving CDW has received much less attention compared to the situation in summer. Yet, the duration of the southward movement lasts longer compared to the northeasterly spreading of the CDW. Further, the concentration of nutrients in this coastal jet is more than an order of magnitude higher than waters further offshore. As a result, the southward flowing CDW becomes an important source of nutrients to the oligotrophic northern South China Sea (SCS)^16,20,22^. But, this southward transport of nutrients has not been adequately studied in a La Niña year when such a transport is expected to be larger because of stronger northeast monsoon. More importantly, the ECS water is relatively rich in nitrogen relative to phosphorus whereas the SCS water is the opposite compared to the optimum Redfield N/P Ratio of 16 for phytoplankton growth^23,24^. Any exchange of waters between the ECS and the SCS through the Taiwan Strait would make the receiving bodies closer to the Redfield Ratio, and potentially enhance biological productivity.

In a normal year, a branch of the Kuroshio enters the SCS via the Luzon Strait and part of the Kuroshio branch veers northward to enter the Taiwan Strait. In the La Niña years such as 2007/2008, the northeast monsoon strengthens. It has been reported that the spring of 2008 sees no Kuroshio water in the southeast Taiwan Strait^25^, and that the salinity is the lowest there during La Niña years because of little intrusion of the high salinity Kuroshio water^26^. Further, Huang et al.^27^ revealed that although the Kuroshio branch did not enter the southeast Taiwan Strait in the spring of 2008 the northward flow increased there because of the large contribution of the SCS waters. It is not known, however, whether the same situation applies in the southwestern Taiwan Strait. Besides, although it may be expected that the southward flowing CDW would strengthen during the La Niña period it is not necessarily so as the above study indicated that the northward flow in the southeastern Taiwan Strait increased when the northeast monsoon strengthened in 2008. What happened is best proven by field data. It is also important to find out whether any Kuroshio-influenced water flows through the Taiwan Strait to enter the ECS in a La Niña year winter/spring.

Here we present shipboard measurements of hydrological parameters in the southwestern ECS to show that even in the mid-spring of 2008, the influence of Kuroshio waters was still minimum in the southern ECS. Instead, a significant amount of the remnants of CDW occupied the near-shore areas of China while further east only SCS waters could be detected at least until off the northeastern tip of Taiwan. Further, satellite images of temperature, Secchi Disk depth (SDD), winds, and sea level anomaly are used to supplement the shipboard data to illustrate the movement of the CDW during the winter/spring of 2007/2008. These are compared with images taken from the same period between 2006 and 2009.

## Study area and methods

**Figure 1:**
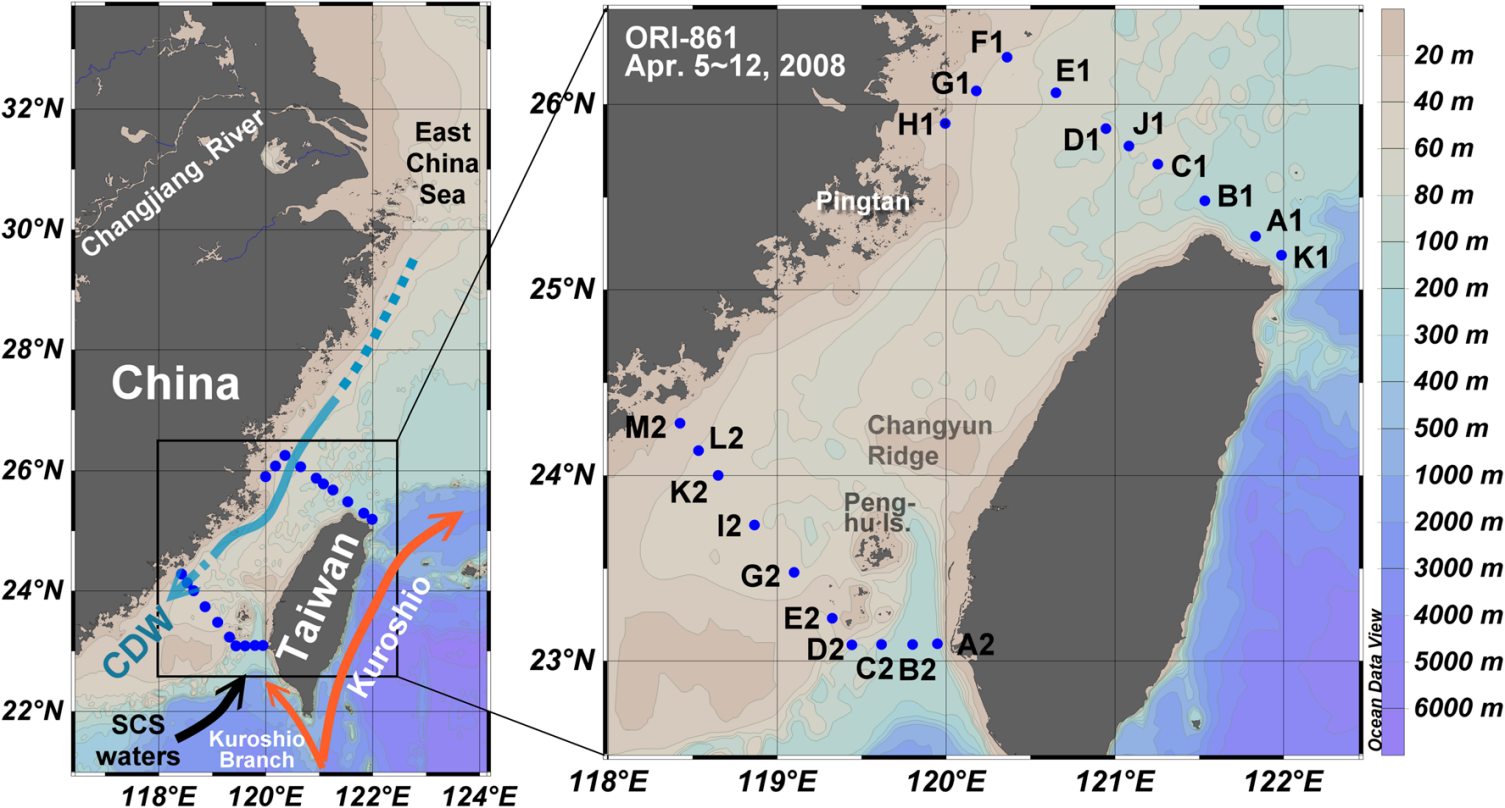
Study area and station locations (map generated by Ocean Data View version 5.2.1.; https://odv.awi.de).

Stations in the southwestern ECS and the southern Taiwan Strait were occupied during cruise 861 of the RV Ocean Researcher I (ORI-861) on 5–12 April 2008. Figure 1 shows the locations of the stations. The shipboard temperature, salinity, and water transparency were determined with a Sea-Bird SBE 911 plus conductivity-temperature-depth (CTD) unit with a transmissometer attached. Seawater samples were collected using a CTD/Rosette sampler fitted with Niskin bottles. Nitrate (NO_3_) plus nitrite (NO_2_) concentrations were measured by the pink azo dye method using a flow injection analyzer with an on-line Cd coil. The precision was about ± 2% at 18 μmol L^−1^ and ± 3% at 1 μmol L^−1^.

The monthly sea surface temperature (SST) was obtained from Aqua/MODIS data provided by the NASA Goddard Space Flight Center, Ocean Biology Processing Group (https://oceancolor.gsfc.nasa.gov/). The MODIS thermal bands at 11 μm were used to derive SST based on a modified version of the nonlinear SST algorithm of Walton et al.^28^, most recently described in Kilpatrick et al.^29^.

To obtain temporal variability of the turbidity data, the daily Aqua/MODIS Level-1 satellite data sets were obtained. The MODIS Level-1 data was processed to Level-2 using the SatDPS system^30^, which used the atmospheric correction algorithm based on the 412 nm band rather than the standard atmospheric correction algorithm in the SeaDAS for highly turbid coastal waters^31^. Based on the spectral remote sensing reflectance derived by the atmospheric correction processing, the sea surface chlorophyll concentration was retrieved using the OC3M algorithm^32,33^, and the total suspended particulate matter concentration (SPM) was retrieved using the local inversion algorithm^34^. Based on the retrieved chlorophyll concentration and SPM concentration, the SDD was then retrieved using the semi-analytical algorithm^35–37^. Finally, the daily SDD data sets were merged to derive the monthly-averaged images.

The Cross-Calibrated Multi-Platform (CCMP) Ocean Surface Wind Velocity Product was obtained from the Remote Sensing Systems (www.remss.com) and have a horizontal resolution of 1/4° by 1/4° and a monthly temporal resolution. The monthly data of multi-satellite-merged sea level anomaly (SLA) were obtained from 2006 to 2009 in a 0.25° × 0.25° regular grid from the Archiving, Validation, and Interpretation of Satellite Oceanographic data (AVISO, http://www.aviso.oceanobs.com) program.

## Results and discussion

**Figure 2:**
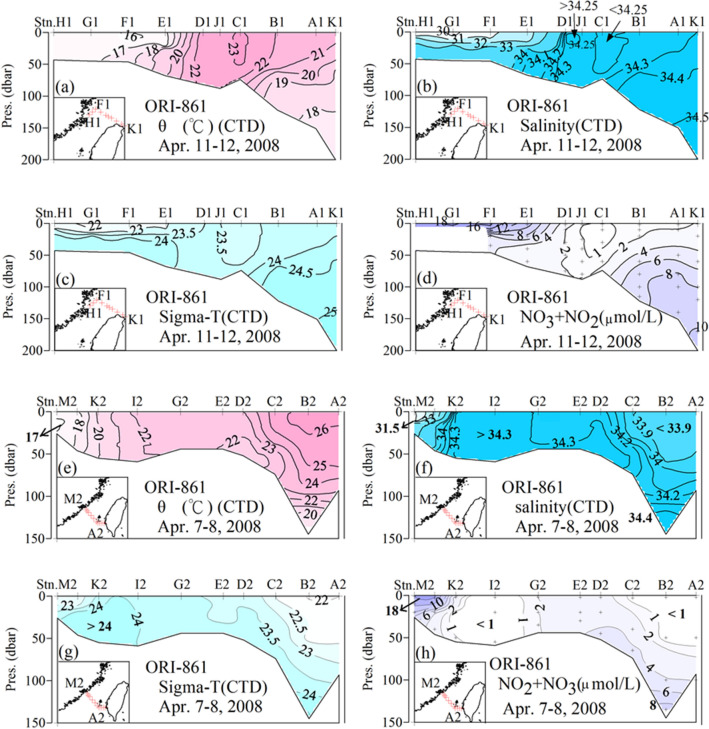
Cross section of (**a**,**e**) θ, (**b**,**f**) S, (**c**,**g**) σ_t_ and (**d**,**h**) NO_3_ + NO_2_ in the southwestern East China Sea and southern Taiwan Strait (map generated by Surfer version 8.0; https://www.goldensoftware.com/products/surfer).

Cross-sections of potential temperature (θ), salinity (S), sigma t (σ_t_), and nitrate plus nitrite (NO_3_ + NO_2_) are displayed in Fig. 2a–h. It can be seen that waters colder than 16 °C with a salinity lower than 30 and a σ_t_ lower than 22 occupy the surface layer of the westernmost stations for the northern transect. Vertical stratification (lower σ_t_ near the surface) is rendered stable by the low salinity near the surface. These cold waters are 1 °C cooler than the coastal waters a few days earlier at the westernmost southern transect, where the salinity was higher at about 31.4. Retreating, remnants of the CDW are most likely responsible for the low temperature and salinity. On the other hand, the surface water NO_3_ + NO_2_ concentrations are higher than 18 μmol L^−1^ near the coast of China, which is more than an order of magnitude higher than surface waters at Stns. J1 and C1 near the middle of the study area. These stations are near a depression at the bottom and display a high temperature (~ 23 °C) and salinity (~ 34.25) but low nutrient (NO_3_ + NO_2_ < 1 μmol L^−1^) core. This is consistent with the same signal found near the bottom depression at Stn. I2 in the western Taiwan Strait in the southern transect. The temperature and salinity there are slightly higher at 24 °C and S = 34.3, but NO_3_ + NO_2_ is equally low at < 1 μmol L^−1^. One explanation for this core of water is that by April a narrow band of waters from the south has already moved northward to our study area following this connecting bottom depression.

**Figure 3:**
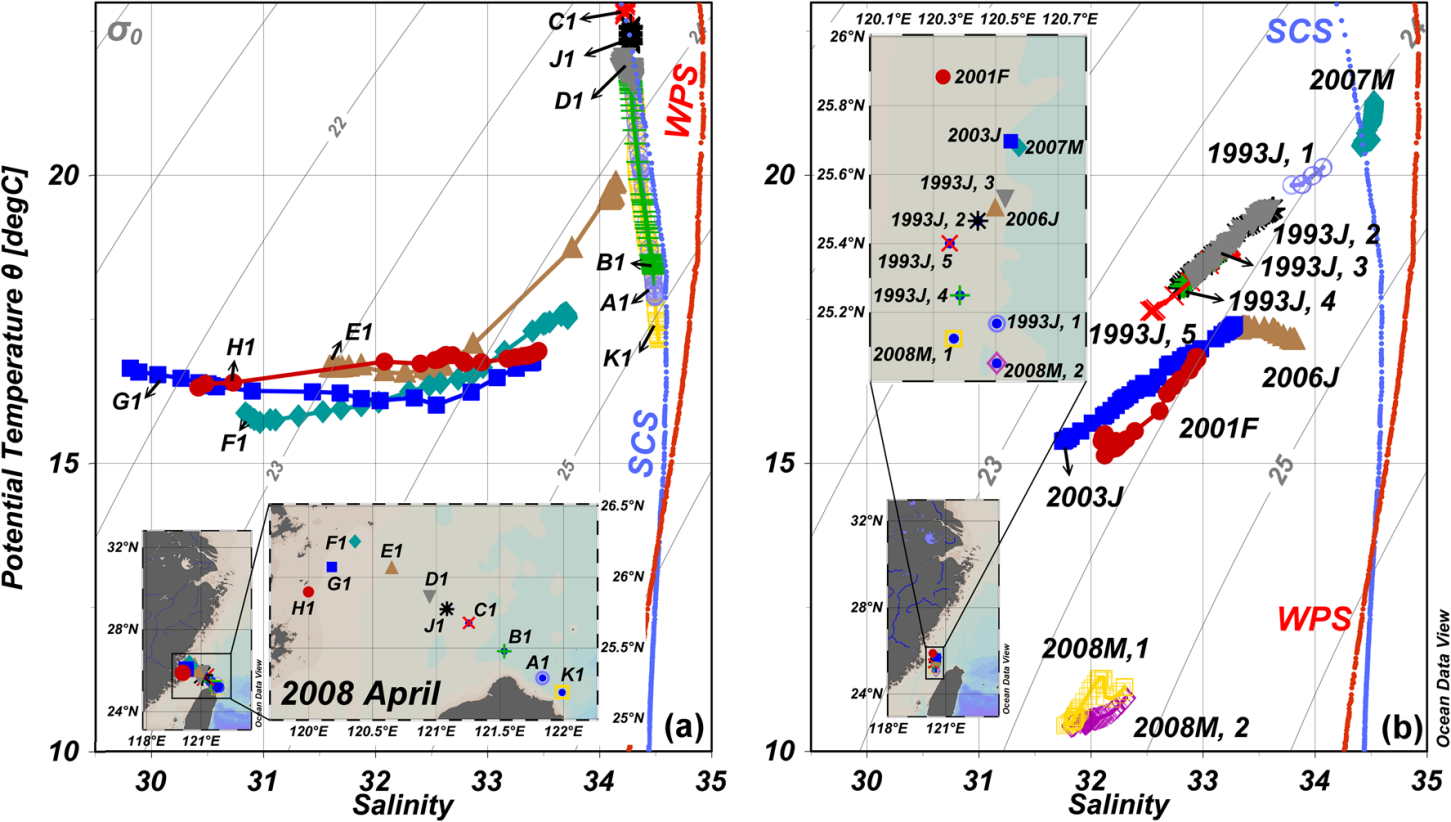
The θ/S plots for the stations in the southwestern East China Sea: (**a**) ORI-861 data and (**b**) various other winter and spring data near Stn. E1 of the ORI-861 cruise (Station is identified by year and month: e.g., 2007 M denotes March, 2007; map generated by Ocean Data View version 5.2.1.; https://odv.awi.de).

It is interesting to note, however, that the θ and S ranges are very small at Stns. J1 and D1, and fall exactly on the same θ/S line found at all stations east of Stn. J1 (Fig. 3a). It is possible that the subsurface Kuroshio waters upwelled off northeastern Taiwan, peaking at Stn. K1 or just east of it. Subsequently, the upwelled waters downwelled towards the west, then upwelled again or became mixed vertically near Stns. J1 and D1. Such a pattern has been reported in the study area in summer^38^, but not in other seasons until recently^39^. As a result, the θ/S pattern for Stns. J1 and D1 falls on the same θ/S line as Stns. C1, B1, A1, and K1. This is the same pattern as Stns. A2–G2 found in the southern transect^25^ (Fig. 2e–h).

Important to point out is that the western side of the Kuroshio is comprised of the outflow from the SCS^40–42^. As a result, whether waters at Stns. J1 and C1 are from the southern Taiwan Strait or are from a branch of the Kuroshio which has circumvented the northern tip of Taiwan, the θ/S patterns are indistinguishable. Of note is that the high density (σ_t_ > 25) water found at the bottom of the easternmost station in the northern transect (Fig. 2c) is much higher than any in the southern transect (Fig. 2g). This is another proof that the high-density water comes from the Kuroshio after circumventing the northern tip of Taiwan. Having said the above, however, albeit the shipboard data collected in April reflect the history of the waters found at the time, it is a snapshot nonetheless. Here we look at consecutive satellite images to help to elucidate the flow patterns before our cruise.

**Figure 4:**
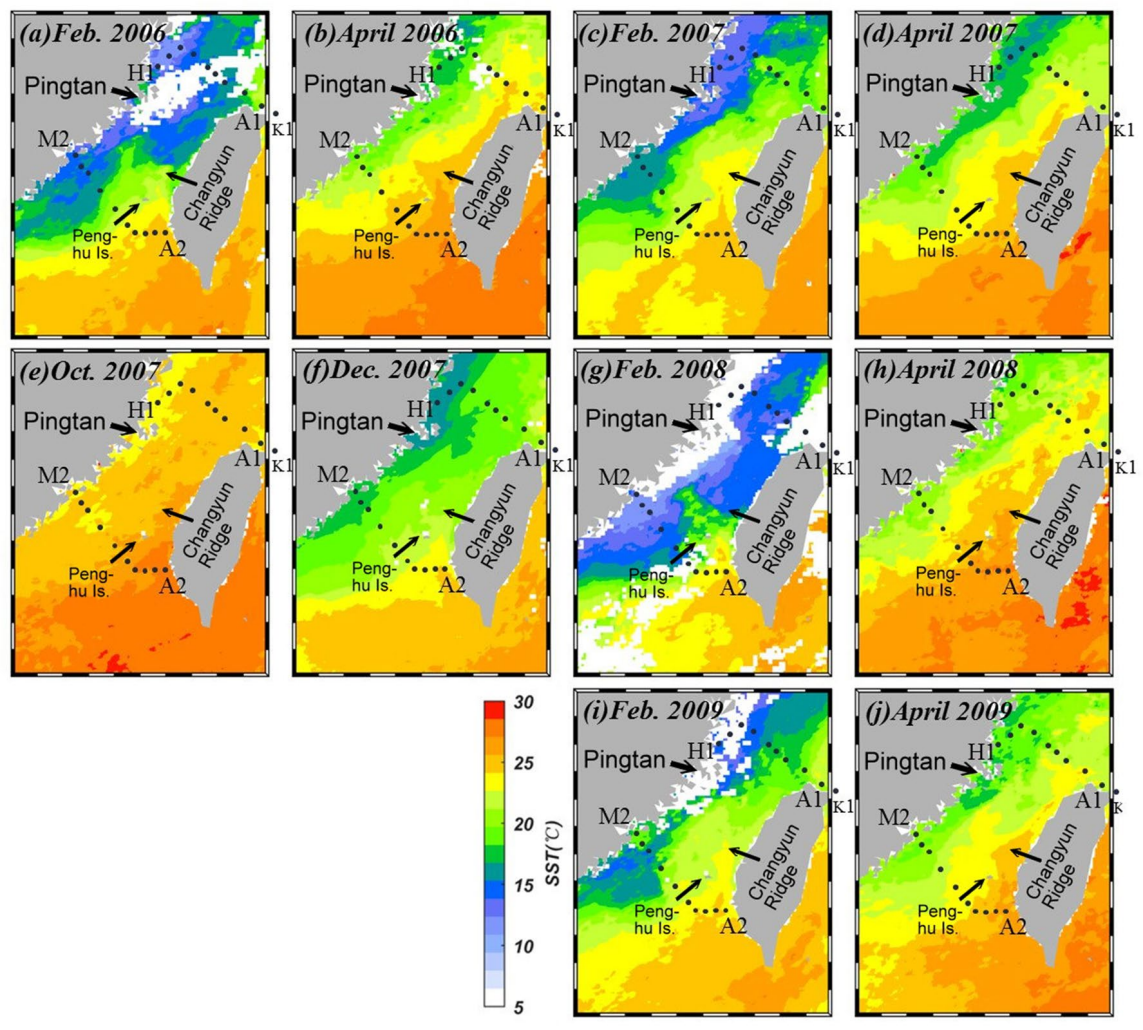
Mean monthly SST in (**a**) Feb. 2006, (**b**) April 2006, (**c**) Feb. 2007, (**d**) April 2007, (**e**) Oct. 2007, (**f**) Dec. 2007, (**g**) Feb. 2008, (**h**) April 2008, (**i**) Feb. 2009 and (**j**) April 2009 in the vicinity of the study area (map generated by using the m_map toolbox in MATLAB 2019a; https://www.mathworks.com/).

Figure 4a–j shows the mean satellite SST in the vicinity of the study area. It was still relatively warm in Oct. 2007 (Fig. 4e) but the ECS was already generally cooler than the SCS and the coastal region off China was generally cooler than offshore areas, especially compared with the Kuroshio region. By Dec. 2007, the temperature in the southwestern East China Sea and most of the Taiwan Strait was relatively cool compared with the SCS and the Kuroshio region (Fig. 4f). A branch of the Kuroshio and the SCS water occupied the southeastern Taiwan Strait but waters cooler than 20 °C, evidently from the north, seemed to have pushed the warmer waters to as far south as Penghu Is. in the southeastern Taiwan Strait. The wind-induced blocking of these warm waters relaxed in late spring, and by April 2008 the warm waters again flew through the eastern Taiwan Strait (Fig. 4h). Figure 4c,i shows that warm waters extended farther north in Feb. 2007 and Feb. 2009 during non-La Niña years. During the La Niña Feb. of 2008, however, cool waters west of Penghu Is. extended farther southward along the coast of China (Fig. 4g). Note waters with temperatures in the lower teens reached the southwestern Taiwan Strait by this time. With cold waters occupying almost the entire Taiwan Strait, the northward movement of the Kuroshio branch and the SCS water was halted, and warm waters were confined to only a small area southeast of the Taiwan Strait. The wind-induced blocking of these warm waters relaxed in late spring, and by April 2008 the warm waters again flew through the eastern Taiwan Strait (Fig. 4h).

By Feb. 2008 seawaters with temperatures in the teens have moved further southwestward along the coast of China. Part of the cold water seemed to have turned toward Taiwan near Pingtan Is. and occupied the entire northern Taiwan Strait. Satellite images indicate that Feb. is the coldest month in the Taiwan Strait. No warm Kuroshio or SCS waters are evident in the Taiwan Strait except off southwestern Taiwan (Fig. 4g). Indeed, the La Niña Feb. of 2008 was exceptionally cold when tons of fish were killed by the cold water around Penghu Is.^43–45^. Cross-strait front and eastward flow of the cold CDW in winter/spring are known to occur^20,46,47^ but it was rare that the cold water reached Penghu Is.

By April 2008 warmer waters have already flowed from the northern SCS to the ECS through the Taiwan Strait (Fig. 4h). Cold waters along the coast of China have by now also been pushed back northward. Waters with temperatures in the low teens have now largely retreated to the coast north of 26° N and Stns. H1, G1, F1, and E1 recorded temperatures around 16, 17 °C (Fig. 2a).

The warmest water found at Stn. C1 in our northern transect is, therefore, originated from the southern Taiwan Strait. This water has a very low NO_3_ + NO_2_ value (Fig. 2d) so it lends support that this water is from the south instead of the retreating, remnant, ECS water. On the other hand, the cooler waters west of Stn. D1 have NO_3_ + NO_2_ concentrations as high as 18 μmol L^−1^ at the surface. Since this high value is much higher than bottom waters found anywhere in our northern cross-section (Fig. 2d) it can not come from upwelling. The only logical explanation is that it is the retreating CDW from the 2007/2008 winter.

Figure 4a,c,i shows the satellite SST images collected for the normal Feb. of 2006, 2007 and 2009. Waters in the ECS were much colder in the La Niña Feb. of 2008 (Fig. 4g), the northern SCS water was also cooler in 2008. Because of the stronger northeast monsoon, cold waters extended farther south along the coast of China in 2008. Figure 3b shows the θ/S plot of winter and spring data from various cruises near Stn. E1 of our study. Indeed, the coldest waters were found in 2008. These waters were among the freshest, in accordance with the notion that the CDW extended southward the most in 2008.

**Figure 5:**
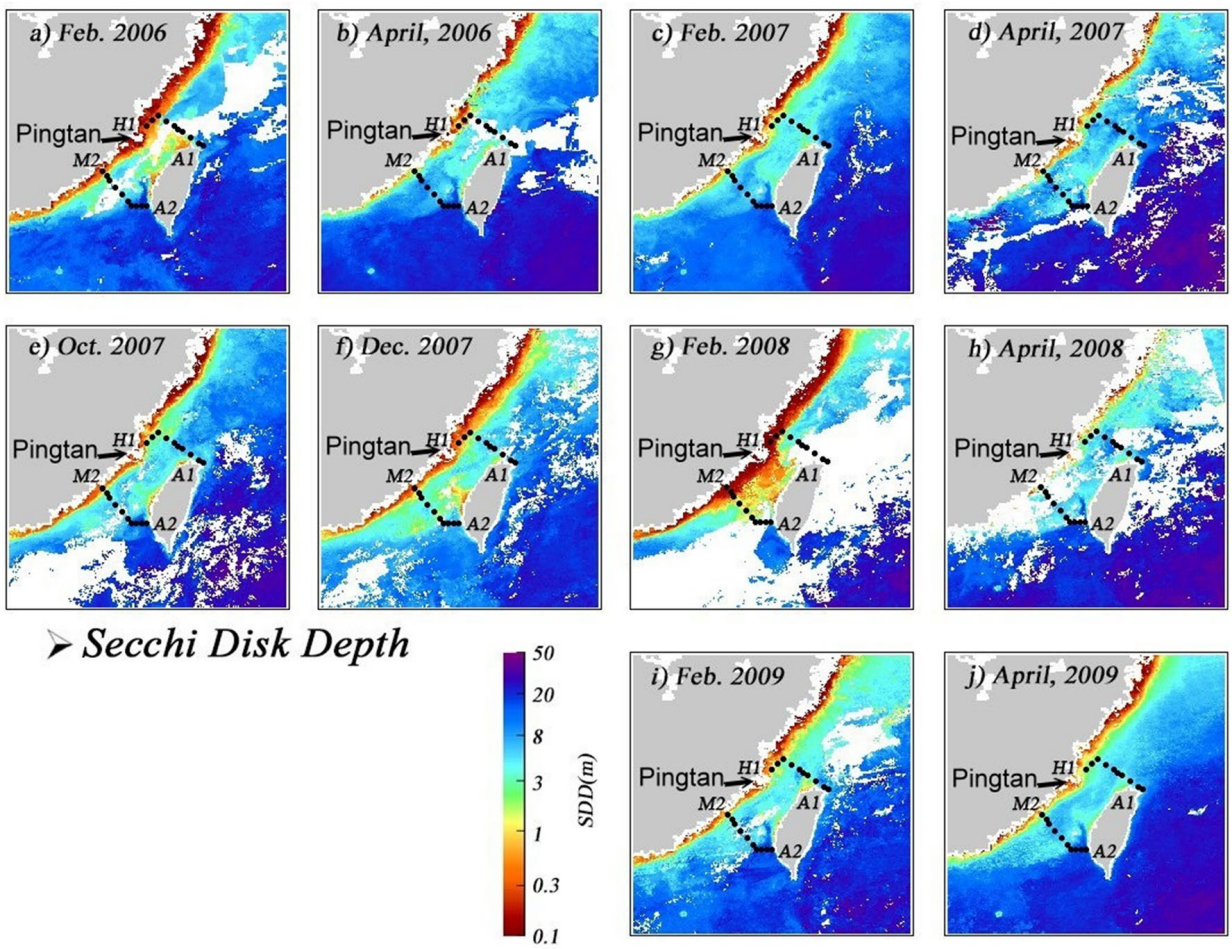
Mean monthly satellite Secchi Disk depth in (**a**) Feb. 2006, (**b**) April 2006, (**c**) Feb. 2007, (**d**) April 2007, (**e**) Oct. 2007, (**f**) Dec. 2007, (**g**) Feb. 2008, (**h**) April 2008, (**i**) Feb. 2009 and (**j**) April 2009 in the vicinity of the study area (map generated by using the m_map toolbox in MATLAB 2019a; https://www.mathworks.com/).

Compared to the SST the water transparency is a much more useful tool in identifying coastal waters in seasons when waters are uniformly warm in general^48^. The satellite Secchi Disk depth images for the months of interest between Feb. 2006 and April 2009 are shown in Fig. 5a–j. Starting from Oct. 2007, half a year before our cruise (Fig. 5e) a band of high turbidity water exists along the coast of China when the SST still does not show much contrast across the Taiwan Strait. A narrower band of turbid water also exists off the western coast of central Taiwan. In the Taiwan St. proper is less turbid water (shown in light blue and green in Fig. 5e). Kuroshio and SCS waters were very clear, as shown by blue in Fig. 5e. The low turbidity water can be seen to extend northward in the southeastern Taiwan Strait but it is forced to turn toward the west south of the Changyun Ridge. It then turned northward again west of the ridge and continued northeastward following the depression in the northeastern Taiwan Strait.

The transparency image in Dec. 2007 (Fig. 5f) is similar to the Oct. image with all features mentioned above intact, except that the high turbidity coastal bands expanded in width and that the turbidity was high on top of the Changyun Ridge. By Feb. 2008 the turbid band along the coast of China expanded even more (Fig. 5g). Similar to the SST image (Fig. 4g) a branch of the high turbidity water veered toward Taiwan and the northward-flowing clean, warm water can no longer be found in the northern Taiwan Strait.

By April 2008, however, the high turbidity coverage was greatly reduced in size (Fig. 5h). Although there was much cloud cover so the transparency image was not very good it can still be seen that a jet of the clean water has penetrated northward east of the Penghu Is. and turned westward south of the Changyun Ridge. Traces of low turbidity water can be seen following the depression in the northern Taiwan Strait and extended to the southern ECS. These are in accordance with the satellite SST image (Fig. 4h) and our shipboard observations. Although it is difficult to tell from the difference in color, the water transparency in Feb., and April of 2006, 2007, and 2009 along the coast of China (Fig. 5a,d,i, j) were more than twice that of the Feb. 2008 values (Fig. 5g). It is easy to tell, however, that the eastward penetration of the high turbidity water found in Feb. 2008 was absent in Feb. 2006, 2007, and 2009. Again, this is perhaps because of the stronger northeast monsoon in the La Niña year of 2008.

**Figure 6:**
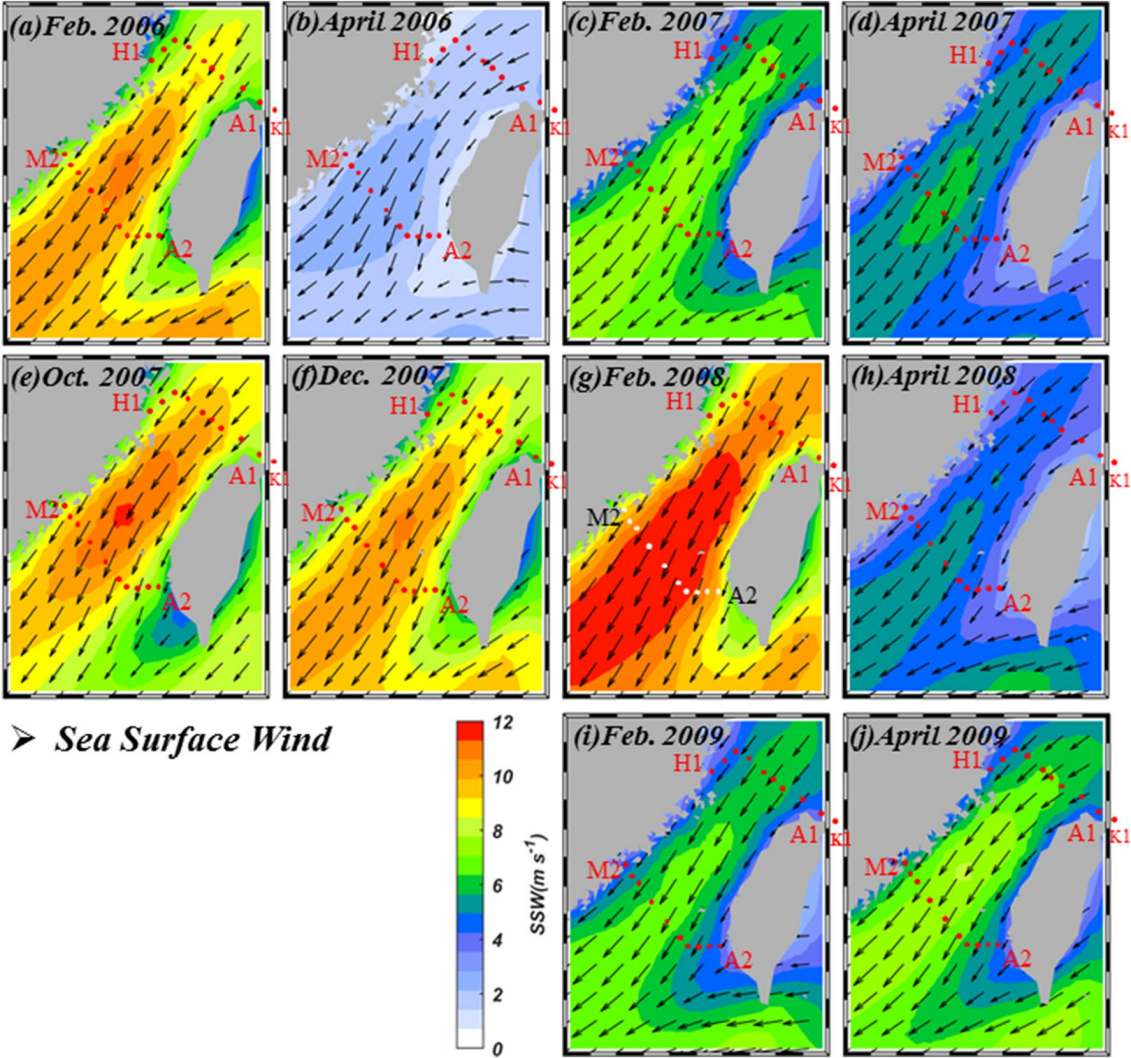
Mean sea surface wind in (**a**) Feb. 2006, (**b**) April 2006, (**c**) Feb. 2007, (**d**) April 2007, (**e**) Oct. 2007, (**f**) Dec. 2007, (**g**) Feb. 2008, (**h**) April 2008, (**i**) Feb. 2009 and (**j**) April 2009 in the vicinity of the study area (map generated by using the m_map toolbox in MATLAB 2019a; https://www.mathworks.com/).

**Figure 7:**
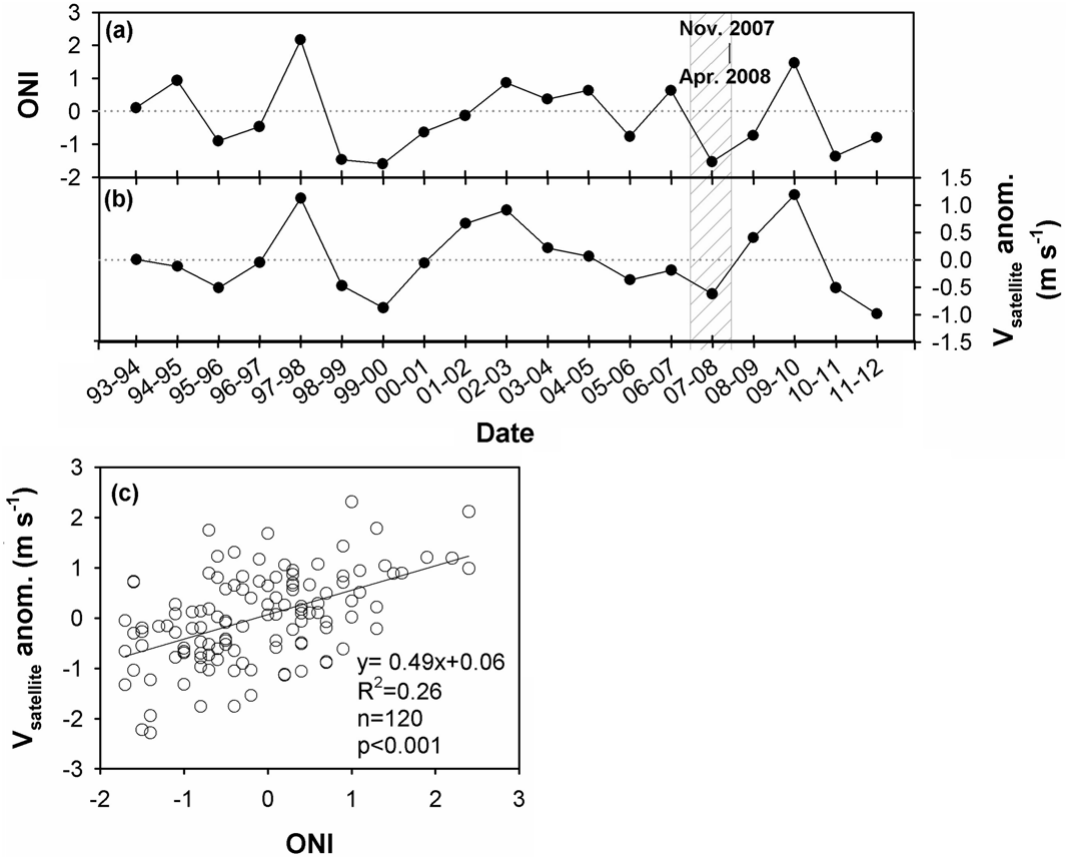
The (**a**) Oceanic Niño Index, (**b**) wind anomaly between Nov. and April from 1993 to 2012, and (**c**) the wind anomaly plotted against ONI.

Figure 6a–j shows the sea surface wind in the study area. Without going into details it is immediately apparent that the northeast winds were the strongest in Feb. 2008 compared with those found in Oct. 2007, Dec. 2007 and April 2008. The Feb. 2008 winds were also the highest compared with the same period in the non-La Niña years of 2006, 2007, and 2009. In addition, we also related the Oceanic Niño Index (ONI; Fig. 7a) with the average v vector of northeast (Fig. 7b) winds between Nov. and April from 1993 to 2012. The ONI was very negative, signaling La Niña, in the fall-spring of 2007/2008. Correspondingly, the northeast winds were strong as indicated by the negative satellite wind anomaly. Figure 7c presents the monthly v vector of the satellite wind anomaly with the monthly ONI. The correlation is highly positive with a *p* < 0.001.

**Figure 8:**
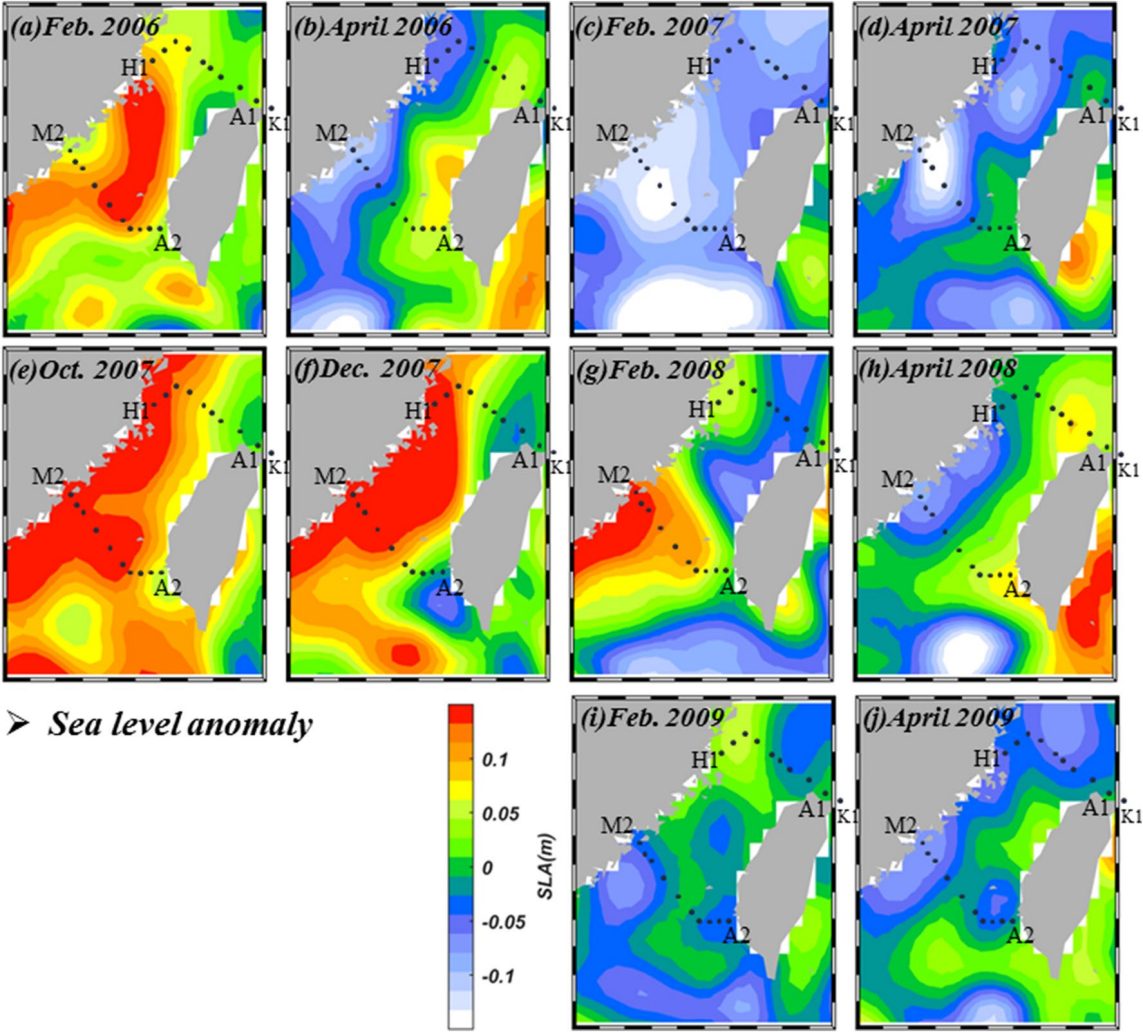
Monthly mean sea level anomaly in (**a**) Feb. 2006, (**b**) April 2006, (**c**) Feb. 2007, (**d**) April 2007, (**e**) Oct. 2007, (**f**) Dec. 2007, (**g**) Feb. 2008, (**h**) April 2008, (**i**) Feb. 2009 and (**j**) April 2009 in the vicinity of the study area (map generated by using the m_map toolbox in MATLAB 2019a; https://www.mathworks.com/).

**Figure 9:**
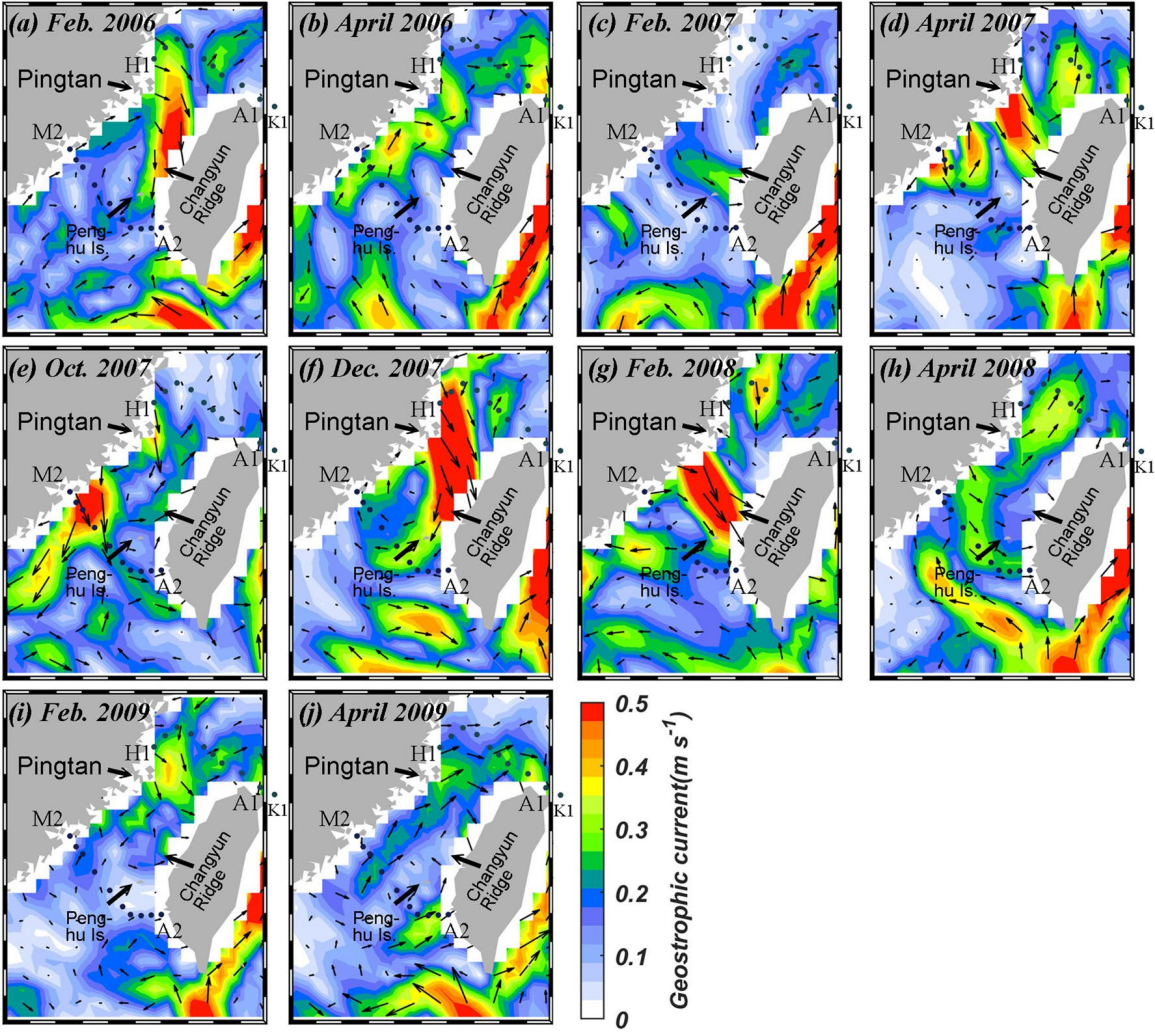
Monthly mean AVISO geostrophic current in (**a**) Feb. 2006, (**b**) April 2006, (**c**) Feb. 2007, (**d**) April 2007, (**e**) Oct. 2007, (**f**) Dec. 2007, (**g**) Feb. 2008, (**h**) April 2008, (**i**) Feb. 2009 and (**j**) April 2009 in the vicinity of the study area (map generated by using the m_map toolbox in MATLAB 2019a; https://www.mathworks.com/).

Figure 8a–j presents the monthly mean SLA images in the vicinity of the study area. When the northeast wind is significant (Fig. 7a,e–g), the Ekman transport induced by the wind generally results in a higher SLA to the west of the Taiwan Strait (Fig. 8a,e,f). In Feb. 2008, however, the sea surface in the central Taiwan Strait tilted downward toward the southeast, indicating a cross-strait flow from the China coast in the northwest to the Penghu Is. in the southeast (Fig. 8g). This is consistent with the SST image in Fig. 4g, as described before. On the other hand, when the monsoon is weak, as in April of 2006, 2007, 2008 and 2009 higher SLA is near the eastern Taiwan Strait which implies a northward flow in the Strait (Fig. 8b,d,h,j). Figure 9 shows the mean AVISO geostrophic current in the study area. It is apparent that the currents were much stronger in the La Niña Feb. of 2008 compared to those in 2006, 2007 and 2009.

For wanting of CDW fluxes it is not yet possible to calculate the southward flux of nutrients. What is attempted here is to use the consecutive satellite SST images to estimate the velocity of the southward movement of waters in the temperature ranges of 7.5–10 °C, 10–12.5 °C, and 12.5–15 °C (Table 1).

**Table 1:** Southward flux of seawater across 25.5° N and 23° N.

T range	Jan Lat	Feb Lat	Monthly mean velocity ( cm/s)	Width (km) 25.5° N	Width (km) 23° N	Depth (m)	Flux (× 10^9^ m^3^) 25.5° N section	Flux (× 10^9^ m^3^) 23° N section
7.5–10.0	28	24	15.4	50	–	20	399.2	
10.0–12.5	25.5	22.9	10.0	100	–	40	1036.8	
12.5–15	24	22.4	6.2	20	100	20	64.3	321.4
Total							1500.3	321.4

From the average width and depth of each temperature band the fluxes of seawater across 25.5° N and 23° N were calculated between Jan. and Feb. 2008. It is found that a total of about 1.5 × 10^12^ m^3^ and 0.3 × 10^12^ m^3^ seawater crossed 25.5° N and 23° N, respectively. Assuming a NO_3_ + NO_2_ concentration of 15 μmol L^−1^ the fluxes were, 2.3 × 10^10^ mol and 0.5 × 10^10^ mol, respectively, across these two cross-sections. Note that although the above calculation is only semi-quantitative these amounts are substantial compared which the annual N flux of 10 × 10^10^ mol from all rivers that enter the SCS^48,49^. Further, these southward moving nutrients are concentrated on the coast, and thus may contribute to eutrophication, even hypoxia, in coastal zones in the following summer.

## Conclusions

Stations in the southwestern ECS and southern Taiwan Strait were occupied between 7 and 12, April 2008. Waters with a temperature lower than 16 °C, salinity lower than 30 but NO_3_ + NO_2_ higher than 18 μmol L^−1^ were found in the nearshore region off China in the northern transect. Waters of 17 °C, S < 32, and NO_3_ + NO_2_ as high as in the northern transect could also be found off China in the central Taiwan Strait. These were the retreating CDW which had previously moved southward. On the other hand, further offshore were waters with temperature as high as 23 °C, salinity higher than 34.3 but NO_3_ + NO_2_ even lower than 0.5 μmol L^−1^. These are northward flowing waters from the south. Since the southward flowing CDW contained as much as 40 times more NO_3_ + NO_2_ than the northward-flowing water, the ECS, by way of the Changjiang River plume in winter, was an important source of nutrient to the oligotrophic, and particularly N-poor South China Sea.

The influence of the CDW in the La Niña spring of 2008 was stronger than the normal spring of 2006, 2007, and 2009. The θ/S analysis indicates that these warm waters were almost pure SCS water with scantly any contribution from the Kuroshio. This is consistent with the finding of Chen et al.^25^ that the La Niña spring of 2008 found no Kuroshio intrusion in the southeastern Taiwan Strait. To better elucidate the movement of water masses we obtained and analyzed satellite SST, SDD, winds, and SLA images in the vicinity of our study area between Feb. 2006 and April 2009. In the La Niña winter/spring of 2008, the SST image started to show indications of more cooling off the coast of China in Oct. 2007. On the other hand, water transparency was already significantly lower for coastal waters compared with offshore waters. By Dec. 2007 both signals showed the southward movement of cold, turbid CDW whereas the northward movement of waters from the south seems to be limited to areas in southeastern Taiwan Strait.

By Feb. 2008 the winds were the strongest, and part of the southward flowing CDW veered toward Taiwan near Pingtan and occupied the entire northern Taiwan Strait. No water from the south could be detected in the northern Taiwan Strait. This is consistent with the SLA images. In the southern Taiwan Strait, the influence of the CDW also expanded in size, further limiting the scope of the northward-flowing current. At the time of our cruise in early April, the CDW had retreated somewhat and the SCS water had again connected the SCS with the ECS in the eastern Taiwan Strait.
